# Beclin1 haploinsufficiency compromises mesenchymal stem cell-offered cardioprotection against myocardial infarction

**DOI:** 10.1186/s13619-022-00121-y

**Published:** 2022-06-02

**Authors:** Xing Qin, Juanjuan Fei, Yu Duan, Asli F. Ceylan, Fuyang Zhang, Jun Ren

**Affiliations:** 1 Department of Cardiology, Xijing Hospital, Fourth Military Medical University, Xi’an, 710032 Shaanxi China; 2 Department of Cardiology and Shanghai Institute of Cardiovascular Diseases, Zhongshan Hospital Fudan University, Shanghai, 200032 China; 3 Department of Medical Pharmacology, Ankara Yildirim Beyazit University, Faculty of Medicine, Bilkent, Ankara, Turkey; 4 Department of Laboratory Medicine and Pathology, University of Washington, Seattle, WA 98195 USA

**Keywords:** Myocardial infarction, MSCs, Beclin1, Contraction, Apoptosis, Autophagy

## Abstract

Mesenchymal stem cells (MSCs)-based therapy has displayed some promises in ischemia heart diseases although its efficacy may be affected by changes in surrounding environments. This study evaluated the role of autophagy insufficiency using Beclin1 haploinsufficiency (BECN^+/−^) on intra-myocardial MSC transplantation-evoked effect against myocardial infarction. Donor MSCs from C57BL/6 mice were labelled with cell-tracker CM Dil and were delivered into LV free wall adjacent to infarct region in wild-type (WT) and BECN^+/−^ recipient mice following ligation of left main coronary artery (MI-MSCs). Ten days following MI, myocardial function was assessed using echocardiography. Cardiomyocyte contractility and intracellular Ca^2+^ were monitored using cardiomyocytes from the area-at-risk adjacent to infarct. CM-Dil labeled cells were tracked in MSCs recipient mice using fluorescence microscopy. Lectin, Masson trichrome staining and Western blot analysis were employed to determine cardiomyocyte area, scar fibrosis, apoptosis and inflammation. MI insult triggered scar fibrosis, LV chamber dilation, decreased fractional shortening, ejection fraction, cardiomyocyte shortening, maximal velocity of shortening and relengthening as well as prolonged relengthening, which were abrogated or attenuated by MSCs therapy in WT but not BECN^+/−^ mice. MI decreased intracellular Ca^2+^ rise and decay in response to electrical stimuli without affecting resting intracellular Ca^2+^, which were reconciled by MSCs in WT but not BECN^+/−^ mice. MSCs further attenuated MI-induced mitochondrial ultrastructural injury, apoptosis, inflammation and autophagy defects in peri-infarct area in WT but not BECN^+/−^ mice. Collectively, our results suggested that autophagy insufficiency dampened in MSCs-elicited cardioprotection associated with dampened apoptosis and inflammation.

## Background

Myocardial infarction (MI) causes irreversible myocardial damage and remains the main drive for heart failure with limited primary and secondary prevention measures available (Chang et al., 2021; Yang et al., 2019a; Alam et al., 2021). The poor ability of mammalian cardiomyocytes to regenerate and proliferate serves as the major factor for post-MI adverse myocardial remodeling and function (Alam et al., 2021; Liang et al., 2021; Xiong, 2020; Davidson et al., 2021). Ample efforts including drug therapy and percutaneous coronary intervention (PCI) procedures in post-MI care help to lower post MI-associated cardiac morbidity and mortality (Zhou et al., 2018; Bhatt et al., 2022; Zhang & Ren, 2016). Among various available therapeutic options, cell transplantation including delivery of progenitor cells and pluripotent stem cells to the infarct region displays promises in tissue repair and functional recovery (Xiong, 2020; Davidson et al., 2021; Martinez-Falguera et al., 2021). For example, transplantation of stem cells derived from bone marrow [i.e., bone marrow (BM)-derived mesenchymal stem cells (MSCs)] has been suggested to offer proven benefits for myocardial tissue repair and recovery as well as mitigate adverse remodeling in pathological settings including MI (Yamada et al., 2021; Pang et al., 2021). Although MSCs were once considered to be capable of generating new cardiac tissues, later findings did not favor de novo cardiomyocyte regeneration following MSCs delivery (Xiong, 2020; Sharma et al., 2022). More evidence suggested that the soil and environment surrounding cardiomyocytes play an important role in the engraftment and retention rates of MSCs, as well as efficacy of stem cell therapy (Sharma et al., 2022; Yang et al., 2018; Zhang et al., 2016). Myocardial stress such as ischemia and energy deprivation usually creates a rather hostile microenvironment with paramount oxidative stress, poor nutrients, and oxygen dearth, prompting injury and poor survival of delivered stem cells (Sharma et al., 2022). More reports suggested that loss of autophagy, an evolutionarily conserved cell event to remove long-lived or injury cellular components (Zhang et al., 2018), dampened hypoxia tolerance of aged BM-MSCs, supporting the importance of sustaining optimal autophagy levels in MSCs transplantation in aged patients (Yang et al., 2018). To this end, this study was designed to evaluate the role of autophagy insufficiency using Beclin1 haploinsufficiency on MSCs-induced beneficial effect against post-MI injury. Given that stem cell therapy may execute its beneficial response through regulation of apoptosis and inflammation (Liang et al., 2021; Davidson et al., 2021; Parizadeh et al., 2019; Ouyang & Wei, 2021), levels of apoptosis and inflammation were monitored in post-MI hearts with or without MSCs transplantation. CM-Dil labelled MSCs were transplanted into recipient hearts immediately after ligation of left coronary artery. Ten days later, scar fibrosis, echocardiographic and morphological properties were evaluated in post-MI murine hearts with or without MSCs transplantation in both wild-type (WT) and Beclin1 (BECN^+/−^) haploinsufficiency mice. Cardiomyocyte mechanical properties were also examined in cardiomyocytes from the area-at-risk adjacent to infarct.

## Results

### Effect of MSCs transplantation on myocardial fibrosis

**Fig. 1:**
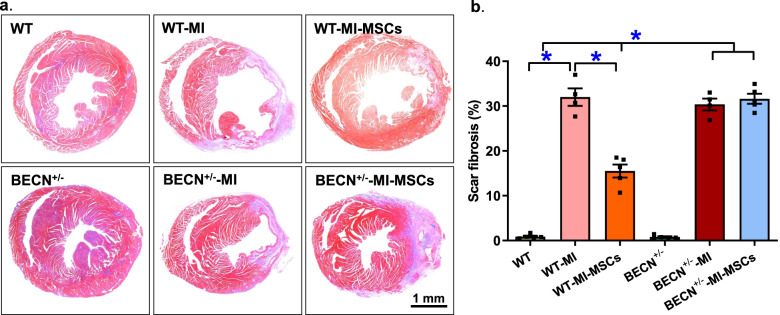
Histological analysis of scar fibrosis using Masson’s trichrome staining in murine hearts from WT and BECN^+/−^ mice following MI with or without a 10-day MSCs transplantation. **a** Representative histological images of Masson trichrome staining (Scale bar = 1 mm); and **b** Quantitated data of scar fibrosis. Mean ± SEM, *n* = 5 mice per group, * *p* < 0.05 between indicated groups

To determine possible effect of MSCs transplantation on myocardial scar fibrosis in post-MI mice, age-matched WT and BECN^+/−^ mice were randomly assigned to untreated, MI and MI-MSCs groups. Ten days following MI and MSCs transplantation, myocardial scar fibrosis was assessed using Masson Trichrome staining. Our data revealed that MI significantly evoked an increase in myocardial fibrotic area (denoting larger infarct) in a comparable manner in both WT and BECN^+/−^ mice with little effect from Beclin1 haploinsufficiency itself (in the absence of MI challenge). Interestingly, MSCs overtly reduced or limited MI-induced myocardial scar fibrosis although such effect was abolished in ECN^+/−^ mice (Fig. 1a-b).

### Cardiomyocyte area and verification of MSCs transplantation in border zone myocardium

**Fig. 2:**
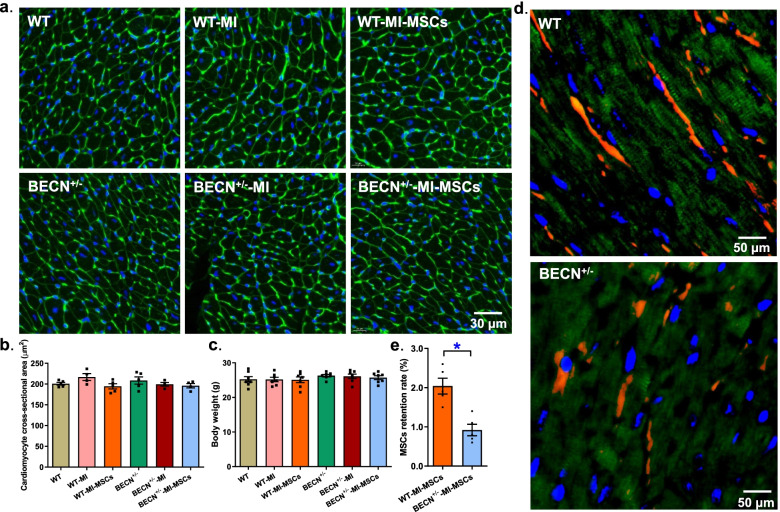
Cardiomyocyte area from WT and BECN^+/−^ mouse hearts following MI with or without MSCs transplantation and illustration of the cell-tracker CM-Dil-positive heart slices from MI border zones 10 days following MSCs transplantation. **a** Representative Lectin staining of cardiomyocyte area; **b** Pooled data of cardiomyocyte area; **c** Body weight; **d** Heart tissues depicting CM-DiI-positive cells (green: troponin T; red: CM-Dil; and blue: DAPI); and **e** MSCs retention rate in WT and BECN^+/−^ mice (rate was determined as area of red CM-Dil fluorescence normalized to the green troponin T-positive area). Mean ± SEM, *n* = 4–7 mice per group, * *p* < 0.05 between indicated groups

Next, we evaluated cardiomyocyte cross-sectional area and tracking of MSCs implantation using Lectin staining and cell-tracker CM-Dil, respectively. Using the same aforementioned group design, neither MI not MSCs transplantation overtly affected cardiomyocyte cross-sectional area in WT or BECN^+/−^ mice (Fig. 2a-b). Body weight was not influenced by MI, MSCs transplantation or Beclin1 haploinsufficiency (Fig. 2c). To track the engraftment and retention of MSCs transplanted to myocardial infarct border zones, slides from myocardium were performed at every 15 μm of tissues away from border zones and were later visualized using a fluorescent microscopy. Figure 2d displayed presence of the CM-Dil-positive MSCs cells in the infarct border zone as shown by red fluorescence. Determination of MSCs retention rate noted a significant drop in MSCs retention rate in BECN^+/−^ group 10 days after MSCs implantation (Fig. 2e).

### Echocardiographic assessment

**Fig. 3:**
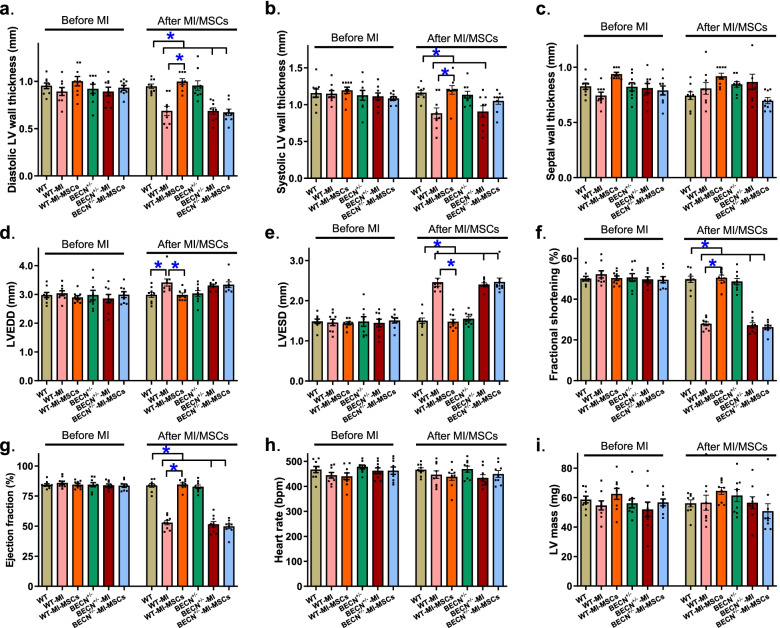
Baseline and post-MI echocardiographic properties of murine hearts from WT and BECN^+/−^ mice immediately before and 10 days following MI procedure with or without MSCs transplantation. **a** Diastolic left ventricular (LV) wall thickness; **b** Systolic LV wall thickness; **c** Septal thickness; **d** LV end diastolic diameter (LV EDD); **e** LV end systolic diameter (LV ESD); **f** Fractional shortening; **g** Ejection fraction; **h** Heart rate; and **i** LV mass. Mean ± SEM, *n* = 9 mice per group, * *p* < 0.05 between indicated groups

To determine the possible effect of MSCs transplantation on MI-induced myocardial injury, age-matched WT and BECN^+/−^ mice were randomly assigned to untreated, MI and MI-MSCs groups. Echocardiographic indices were acquired immediately before MI and/or MSCs procedures At the end of the 10-day MI and/or MSCs procedures, echocardiographic assessment was assessed again in WT and BECN^+/−^ mice to discern myocardial geometry and function. Our data shown in Fig. 3 displayed comparable echocardiographic parameters in all 6 mouse groups prior to the MI or MSCs transplantation procedures. MI significantly evoked a rise in LV EDD and LV ESD as well as decreased LV wall thickness (diastolic and systolic), fractional shortening and ejection fraction without affecting septal thickness, heart rate and LV mass, the effects of which were overtly attenuated or ablated by MSCs transplantation. Although Beclin1 haploinsufficiency did not elicit any discernable effect on baseline or MI-evoked changes in echocardiographic indices (MI-induced rise in LV EDD failed to reach statistical significance in BECN^+/−^ mice), it removed MSCs-induced beneficial effects against MI (Fig. 3).

### Baseline mechanical and intracellular Ca^2+^ properties of cardiomyocytes

**Fig. 4:**
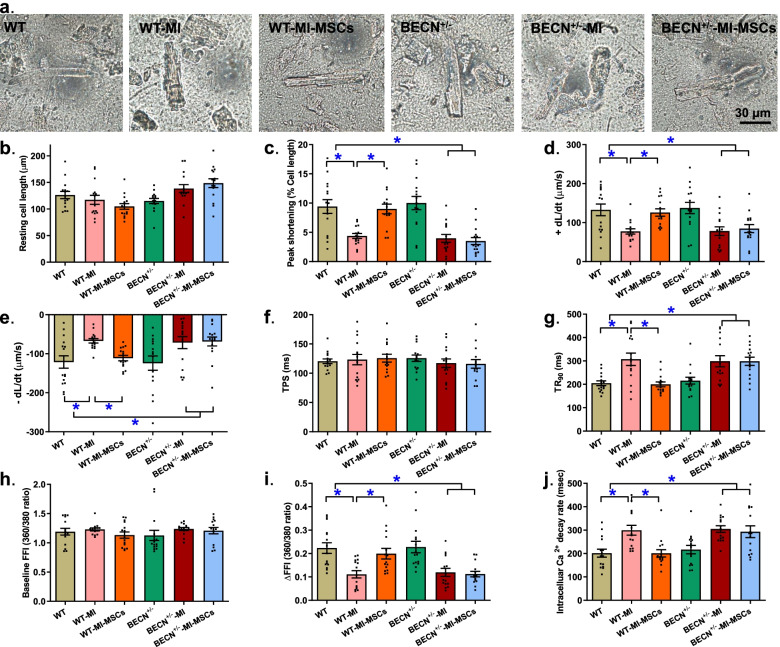
Cardiomyocyte contractile and intracellular Ca^2+^ properties from area-at-risk from WT and BECN^+/−^ mouse hearts following MI with or without MSCs transplantation. **a** Light microscopic images of freshly isolated cardiomyocytes: **b** Resting cell length; **c** Peak shortening (normalized to resting cell length); **d** Maximal velocity of shortening (+ dL/dt); **e** Maximal velocity of relengthening (− dL/dt); **f** Time-to-peak shortening (TPS); **g** Time-to-90% relengthening (TR_90_); **h** Baseline intracellular Ca^2+^ fura-2 fluorescence intensity (FFI); **i** Electrically-stimulated increase in fura-2 fluorescence intensity (ΔFFI); and **j** Intracellular Ca^2+^ transient decay rate. Mean ± SEM, *n* = 15 cardiomyocytes per group, * *p* < 0.05 between indicated groups

MI insult significantly decreased cardiomyocyte peak shortening (PS), maximal velocity of shortening/relengthening (± dL/dt) and prolonged time-to-90% relengthening (TR_90_) without affecting time-to-PS (TPS) in cardiomyocytes isolated from area-at-risk, in a comparable manner, in WT and BECN^+/−^ mice. Transplantation of MSCs overtly ameliorated or negated MI-induced cardiomyocyte dysfunction in WT but not BECN^+/−^ mice (Fig. 4a-g). To explore any possible mechanisms of action behind Beclin1 haploinsufficiency-induced effect against MSCs-induced myocardial and cardiomyocyte mechanical responses, intracellular Ca^2+^ handling was monitored in cardiomyocytes from the area-at-risk in MI-challenged and MSC-delivered WT and BECN^+/−^ mice. Our data revealed that MI drastically decreased electrically-induced rise of intracellular Ca^2+^ levels and prolonged intracellular Ca^2+^ clearing without affecting resting intracellular Ca^2+^ levels, in a comparable manner, in both WT and BECN^+/−^ mice. Reminiscent of its effect on cell shortening, transplantation of MSCs significantly attenuated or negated MI-induced anomalies in intracellular Ca^2+^ handling in WT but not BECN^+/−^ mice (Fig. 4h-j).

### Ultrastructural examination and mitochondrial integrity

**Fig. 5:**
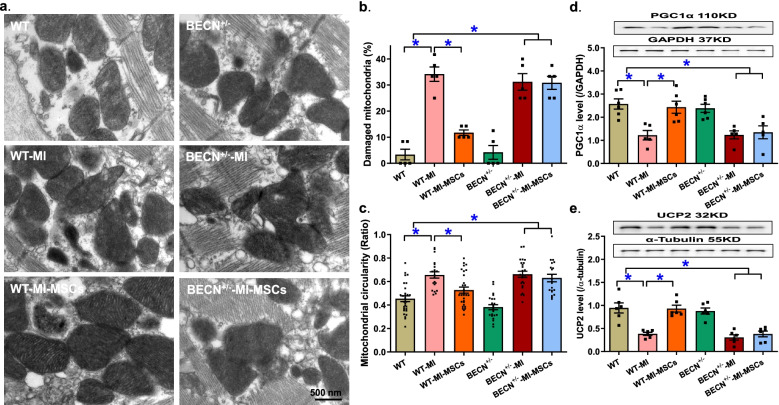
Ultrastructural myocardial and mitochondrial properties of area-at-risk myocardium from WT and BECN^+/−^ mice following MI with or without MSCs transplantation. **a** Representative TEM ultrastructural images from all mouse groups; **b** Percentage of damaged mitochondria; **c** mitochondrial circularity (short axis/long axis); **d** PGC1α levels; and **e** UCP2 levels. Insets: Representative immunoblots depicting PGC1α and UCP2 using specific antibodies (GAPDH or α-Tubulin as loading controls); Mean ± SEM, *n* = 5–6 mice or 19–29 mitochondria from 5 TEM images (panel c) per group, * *p* < 0.05 between indicated groups

Given the prominent role of mitochondrial integrity in the maintenance of cardiac hemostasis (Chang et al., 2021; Ren et al., 2021), mitochondrial morphology, mitochondrial injury and content were examined in the aforementioned 6 experimental mouse groups. TEM ultrastructural analysis revealed pronounced cytoarchitectural aberrations including loss or fragmentation of mitochondrial cristae, mitochondrial swelling, and distortion of sarcomeres as a consequence of MI insult, the effects of which were moderately lessened or reversed in MSCs-transplanted mice. In particular, percent of damaged mitochondria and mitochondrial circularity were overtly elevated in MI challenged mice in a comparable manner in WT mice, the effect of which was significantly alleviated by MSCs transplantation. Although Beclin1 haploinsufficiency failed to elicit any notable response on myocardial ultrastructure at basal or MI-challenged conditions, it effectively nullified MSCs-offered benefit against MI infarction (Fig. 5a-c). This is in line with immunoblot findings of mitochondrial biogenesis or energy metabolism proteins PGC1α and UCP2. Western blot analysis revealed that 10-day MI insult overtly downregulated levels of the mitochondrial biogenesis cofactor PGC1α and mitochondrial uncoupling protein UCP2, the effect of which was ablated by MSCs-transplantation. Although Beclin1 haploinsufficiency did not elicit any discernable effect on PGC1α and UCP2 under basal or MI-challenged conditions, it obliterated MSCs-offered preservation of PGC1α and UCP2 under MI infarction (Fig. 5d-e).

### Western blot analysis of apoptosis, inflammation and autophagy

**Fig. 6:**
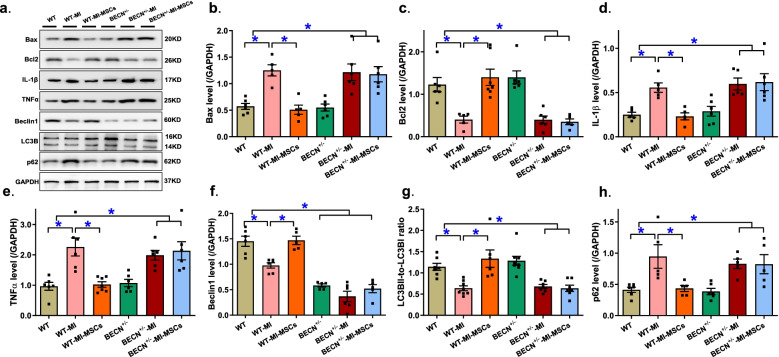
Assessment of apoptosis, inflammation and autophagy in myocardium from area-at-risk from WT and BECN^+/−^ mice following MI procedure with or without MSCs transplantation. **a** Representative immunoblots depicting levels of Bax, Bcl2, IL1β, TNFα, Beclin1, LC3B and p62 using specific antibodies (GAPDH or α-Tubulin as loading controls); **b** Bax levels; **c** Bcl2 levels; **d** IL1 levels; **e** TNFα levels; **f** Beclin1 levels; **g** LC3B ratio; and **h** p62 levels. Mean ± SEM, *n* = 5–7 mice per group, * *p* < 0.05 between indicated groups

Given that MSC transplantation was reported to affect cell death and survival (Davidson et al., 2021; Parizadeh et al., 2019; Broughton et al., 2018), apoptosis, inflammation and autophagy were evaluated in myocardium extracted from the peri-infarct area. Our data demonstrated that MI insult overtly upregulated levels of Bax, IL1β, TNFα, and p62 while downregulating levels of Bcl2, Beclin1, LC3BII-to-LCBI ratio in myocardium from peri-infarct region from post-MI hearts, the effects of which were negated by MSCs transplantation. Although Beclin1 haploinsufficiency failed to elicit notable effect on these apoptosis, inflammatory and autophagy protein markers in basal or MI-stressed conditions (with exception of Beclin1 haploinsufficiency validating the mouse model), it negated MSCs-induced protection against apoptosis, inflammation and dampened autophagy (Fig. 6). These data suggest possible involvement of apoptosis, inflammation and autophagy in MSC2-induced cardioprotection and an obligatory role for autophagy in MSCs-offered benefit against post-infarct injury.

## Discussion

Stem cell therapy has attracted much attention over the past decades in the management of post-MI injury (Xiong, 2020; Parizadeh et al., 2019; Broughton et al., 2018) although little is known with regards to the role of autophagy in MSCs-induced cardioprotection in MI. The salient findings from our current study suggested that deficiency in the autophagy initiating gene Beclin1 greatly dampened MSCs transplantation-offered benefit against post-MI myocardial injury. This result is in line with the findings from previous reports of the beneficial role of autophagy in heart regeneration (Davidson et al., 2021; Xie et al., 2021; Park et al., 2021). Our data revealed that MSC delivery ameliorates MI insult-induced myocardial scar fibrosis, echocardiographic, cardiomyocyte contractile and intracellular Ca^2+^ dysfunction as well as mitochondrial injury accompanied by suppression of apoptosis, inflammation and autophagy defect. We reported for the first time that lack of the autophagy gene Beclin1 nullified MSCs transplantation-evoked benefit against post-MI injury, indicating the possible contribution of autophagy status in MSCs-elicited myocardial protection following MI.

Perhaps the most prominent sequelae of post-infarct injury are the loss of myocardial function and cardiac remodeling including interstitial fibrosis, enlargement of LV chamber, dampened fractional shortening and ejection fraction (Yu et al., 2020; Xu et al., 2021; Yan et al., 2020). This is in line with the findings from our current study where MI evoked myocardial scar fibrosis, abnormal echocardiographic changes including enlarged LV ESD, decreased fractional shortening and ejection fraction with unchanged LV and septal wall thickness, LV EDD, heart rate and LV mass. Evaluation of cardiomyocytes from area-at-risk surrounding the infract noted overtly decreased peak shortening (PS), and maximal velocity of shortening/relengthening (± dL/dt) along with prolonged TR_90_ with little changes in duration of shortening. Our result also reported that MI insult decreased rise of intracellular Ca^2+^ levels and prolonged intracellular Ca^2+^ clearance. These MI-induced morphological, echocardiographic, cardiomyocyte contractile and intracellular Ca^2+^ defects were greatly attenuated or nullified following the 10-day MSCs transplantation, consistent with our previous findings (Yan et al., 2020; Li et al., 2010). Moreover, MSCs transplantation effectively alleviated MI-induced apoptosis, inflammation and autophagy defect, suggesting a possible role for these cellular events in MSCs-offered benefits against post-MI injury.

Perhaps the most intriguing data from our present study is that Beclin1 haploinsufficiency nullified MSCs-offered cardioprotection against post-MI injury. With deficiency in autophagy initiating molecule Beclin1, MSCs delivery were unable to effectively attenuate post-MI injury including defects in myocardial scar fibrosis, echocardiographic, cardiomyocyte contractile and intracellular Ca^2+^ parameters. Along the same line, MSCs-evoked beneficial effects in apoptosis, inflammation and autophagy were also dampened in BECN^+/−^ mice. These findings strongly suggest a seemingly obligatory role for autophagy (or the autophagy initiating gene Beclin1) in MSCs-induced cardioprotection against post-MI injury. Over the past decades, stem cell therapy including embryonic stem cells, MSCs, human cord blood cells, progenitor cells and menstrual blood-derived mesenchymal cells offers cardioprotection against MI-evoked cardiac dysfunction (Alam et al., 2021; Davidson et al., 2021; Yamada et al., 2021). MSCs belong to a type of stem cells with promising therapeutic applications. Cardiac stem cells are rare with one stem cell in every 18,000 cardiomyocytes (Broughton et al., 2018), making MSCs as an excellent choice for myocardial tissue repair (Davidson et al., 2021). Ample evidence depicted that MSCs delivery may benefit post-MI cardiac function (Davidson et al., 2021; Yan et al., 2020; Li et al., 2010). Up-to-date, a number of scenarios have been put forward for MSCs-offered cardioprotection including anti-apoptosis and pro-angiogenesis (de Freitas et al., 2021). More recently, autophagy was shown to positively correlate with metformin-induced heart regeneration in zebrafish and suggests the value of this diabetic drug for amelioration of MI injury (Xie et al., 2021). In addition, inhibition of mammalian target of rapamycin (mTOR) using HY-1685 may also rejuvenate senile human cardiac stem cells through modulating autophagy to improve human cardiac stem cell cell-based myocardial regeneration (Park et al., 2021). These observations denoted the important role for autophagy homeostasis in stem cell-based myocardial repair and regeneration.

Finding from our current study noted a significant decline of MSCs retention in Beclin1 haploinsufficiency mice, suggesting an important role for autophagy in cell survival of MSCs. The viability of implanted stem cells appears to be somewhat low when directly delivered into the infract region due to severe ischemic injury and apoptosis (Broughton et al., 2018). With this in mind, MSCs were delivered directly into the border zone to best improve MSCs cell viability. Indeed, only a small fraction (1–2%) of CM-Dil-positive cells were tracked in the border zone from recipient mouse hearts. The significant decline of MSCs retention in BECN^+/−^ mice indicates the essential role for autophagy status in MSCs cell survival as this prevails for many cell types in the heart (Chang et al., 2021; Davidson et al., 2021). Considering the prominent effects of intramyocardial MSCs transplantation on scar fibrosis, echocardiographic, cardiomyocyte contractile and intracellular Ca^2+^ as well as mitochondrial properties, and the fact that such effects were ablated by Beclin1 haploinsufficiency, it is likely that MSCs therapy improve post-MI injury through inhibition of apoptosis /inflammation in an autophagy-dependent manner, other than the rare cell regenerative ability in post-infarct hearts. Nonetheless, further studies are warranted to discern the precise role of autophagy in particular Beclin1 in MSCs-offered cardioprotection against post-MI injury.

### Experimental limitations

Certain limitations exist for our study. First and foremost, our findings cannot rule out possible direct beneficial effect of autophagy on myocardial tissue repair following MI. Autophagy or mitochondria-selective autophagy may exert protective effects against MI injury (Chang et al., 2021; Yang et al., 2019a; Zhou et al., 2018) or directly serves as a target for stem cell therapy (Davidson et al., 2021). Thus, special caution should be taken for data interpretation. Next, the efficacy of stem cell therapy is often crippled by presence of inadequate paracrine function or environment involving growth factors, extracellular vesicles (e.g., exosomes for transport of proteins, lipids, non-coding RNAs), which all contribute to angiogenesis and myocardial regeneration (Davidson et al., 2021). Further study is warranted for the role of paracrine factors in autophagy-mediated regulation of stem cell therapy.

## Conclusions

Taken together, data from our study indicated that MSC transplantation rescues against post-MI cardiac injury via an autophagy-dependent manner. Beclin1 haploinsufficiency negated MSCs-offered benefits against MI-induced myocardial scar fibrosis, echocardiographic defect and cardiomyocyte mechanical dysfunction in cells from the area-at-risk. This is in line with the observation of changes of apoptosis and inflammation in MSCs-treated WT and BECN^+/−^ mice with MI injury. These findings have shed lights towards a better understanding for the obligatory role of Beclin1 and autophagy in MSCs and possibly stem cell therapy in ischemic heart diseases.

## Methods

### Delivery of CM-Dil-labelled bone marrow mesenchymal stem cells (MSCs)

All experimental procedures were approved by the Institutional Animal Use and Care Committee at the Fourth Military Medical University (Xi’an, China) and was in compliance with the Guide for the Care and Use of Laboratory Animals published by NIH. C57BL/6 J mice were used as donor mice. In brief, six-month-old C57BL/6 J WT or BECN^+/−^ mice were employed as the MSCs recipient or control mice. Beclin1 haploinsufficiency (BECN^+/−^) mice were provided by Prof. Zhenyu Yue from Mount Sinai School of Medicine (New York, NY, USA) with heterozygous deletion of Beclin1 (Liu et al., 2020; Yue et al., 2003). All mice were maintained in a temperature-controlled room under a 12 h/12 h-light/dark with free access to rodent chow and tap water ad libitum. In brief, six-week-old C57BL/6 J donor mice were anesthetized using ketamine/xylazine (3:1, 1.32 mg/kg, i.p.). Bone-marrow plugs of femora and tibiae were flushed with a 22-gauge needle and a syringe containing phosphate-buffered saline solution (PBS). Bone-marrow cells were suspended in PBS and were centrifuged. Cell pellets were resuspended in a MesenCultTM medium supplemented with the Mesenchymal Stem cell Stimulatory Supplements (StemCell Technologies. Vancouver, Canada) prior to cell incubation at 37 °C with 5% CO_2_. Primary cultures of MSC usually reached ~ 90% confluence within 10 days. The fourth-to-fifth passage cells were trypsinized and suspended in PBS prior to treatment with the cell-tracker CM-Dil (C_68_H_105_Cl_2_N_3_O, 5 μM, ThermoFisher) for 20 min in the medium before intramyocardial delivery into the heart. The retention rate of MSCs was observed and calculated based on the area of the CM-Dil positive red fluorescence normalized to the troponin T positive green fluorescence (Yan et al., 2020).

### Myocardial infarction model and cell transplantation

Adult male C57BL/6 J WT and BECN^+/−^mice (6-month-old) were endotracheally intubated using a ventilator. Anesthesia was sustained using the ketamine/xylazine anesthetic/analgesic combination. Thoracotomy was performed and a 6–0 ethilon ligature was tied around left anterior descending artery 4–5 mm above left atrium. Approximately 5 × 10^5^ MSCs were delivered into non-ischemic (border) zones (single injection). Chest was closed prior to weaning of mice from ventilator. Occlusion of left anterior descending coronary artery (LAD) and injection of MSCs were executed surgically with the aid of a Leica dissecting microscope. Border zones were selected for MSC transplantation to maximize graft viability. Ischemic and proapoptotic properties of infarcted region were deemed unsuitable for graft viability. Mice were maintained on a small animal respirator during the thoracotomy, infarct, injection and recovery periods (Yu et al., 2020; Li et al., 2010).

### Masson trichrome staining for scar fibrosis

Mouse hearts were arrested in diastole by injection of 10% potassium chloride after anesthesia. Hearts were excised and placed in paraformaldehyde prior to fixation in paraffin. Myocardial sections (4-μm thick) were stained with Masson trichrome to detect myocardial scar interstitial fibrosis. Images were taken using a Leica microscopy at 5 × objective. Scar fibrosis was calculated as the fraction of light blue–stained area normalized to the total area using the Image J software (Yu et al., 2020).

### Transmission electron microscopy (TEM)

Small cubic pieces of left ventricles ≤1mm^3^ were fixed with 2.5% glutaraldehyde in 0.1 M sodium phosphate (pH 7.4) overnight at 4 °C prior to Epon Araldite embedding. Ultrathin sections (50 nm) were sliced using an ultramicrotome (Ultracut E, Leica), and were stained with uranyl acetate and lead citrate. The specimens were imaged through a Hitachi H-7000 Electron Microscope (Pleasanton, CA) equipped with a Gatan high resolution digital camera (Wang et al., 2018a).

### Echocardiographic assessment

Cardiac geometry and function were monitored in anesthetized (80 mg/kg ketamine and 12 mg/kg xylazine, i.p.) mice using 2-dimensional guided M-mode echocardiography (Sonos 5500, Phillips Medical Systems, Andover, MD, USA) equipped with a 15–6 MHz linear transducer. Left ventricular (LV) dimensions were recorded and fractional shortening was calculated from LV end-diastolic (EDD) and end-systolic diameters (ESD) using the equation of (LVEDD - LVESD)/LVEDD. Ejection fraction, heart rate and LV mass were derived per previously published protocols (Yang et al., 2019b). Echocardiographic assessment was performed immediately before (to ensure all groups were at a comparable starting point) and 10 days after MI/MSCs procedure.

### Preparation of cryostat section from heart tissue after stem cell transplantation and tracking of MSCs

To track the fate of stem cells 10 days after MSCs transplantation, cryostat sections were cut from snap-frozen heart tissues around the sites of MSCs injection. Myocardial tissue section thickness was 5 μm. All sections were visualized under a fluorescent microscopy. Retention rate of MSCs was determined based on CM-Dil-labelled red fluorescence (Yan et al., 2020; Li et al., 2010).

### Isolation of mouse cardiomyocytes

Following ketamine/xylazine sedation, hearts were removed and perfused with Krebs-Henseleit bicarbonate (KHB) buffer containing (in mM): 118 NaCl, 4.7 KCl, 1.2 MgSO_4_, 1.2 KH_2_PO_4_, 25 NaHCO_3_, 10 HEPES and 11.1 glucose. Hearts were digested with collagenase D for 20 min. Left ventricles were removed and minced. Cardiomyocyte yield was ~ 75% which was not affected by cold exposure or Beclin1 haploinsufficiency. Only rod-shaped myocytes with clear edges were selected for mechanical study (Li et al., 2010).

### Cell shortening/relengthening

Mechanical properties of cardiomyocytes were assessed using an IonOptix™ soft-edge system (IonOptix, Milton, MA, USA). Cardiomyocytes were placed in a chamber mounted on the stage of an Olympus IX-70 microscope and superfused (~ 2 ml/min at 25 °C) with a KHB buffer containing 1 mmol/l CaCl_2_. Myocytes were field stimulated at 0.5 Hz. Cell shortening and relengthening were assessed including peak shortening (PS), time-to-PS (TPS), time-to-90% relengthening (TR_90_) and maximal velocity of shortening/relengthening (± dL/dt) (Wang et al., 2017).

### Intracellular Ca^2+^ transient measurement

A cohort of myocytes was loaded with fura-2/AM (0.5 μM) for 15 min, and fluorescence intensity was recorded with a dual-excitation fluorescence photomultiplier system (Ionoptix). Fluorescence emissions were detected between 480 and 520 nm; qualitative change in fura-2 fluorescence intensity (FFI) was inferred from the fura-2 fluorescence intensity ratio at the two wavelengths (360/380). Fluorescence decay time (single exponential) was calculated as an indicator of intracellular Ca^2+^ clearance. Cells were exposed to light emitted by a 75-W lamp while being stimulated to contract at a frequency of 0.5 Hz (Wang et al., 2017).

### Lectin staining

After anesthesia, hearts were excised and immediately placed in 10% neutral-buffered formalin at room temperature for 24 h after a brief rinse with PBS. The specimens were embedded in paraffin, cut into 5-μm sections and stained with fluorescein isothiocyanate (FITC)-conjugated wheat germ agglutinin. Cardiomyocyte cross-sectional areas were calculated on a digital microscope (× 400) using the Image J (version1.34S) software (Wang et al., 2018b).

### Western blot analysis

Samples were separated on 10% SDS-polyacrylamide gels in a minigel apparatus (Mini-PROTEAN II, Bio-Rad) and transferred to nitrocellulose membranes. The membranes were blocked with 5% milk in TBS-T, and were incubated overnight at 4 °C with anti-PGC1α, anti-UCP2, anti-IL1β, anti-TNFα, anti-Beclin1, anti-LC3B, anti-p62, anti-Bax, and anti-Bcl2 antibodies. After immunoblotting, the film was scanned and intensity of immunoblot bands was detected with a Bio-Rad Calibrated Densitometer. GAPDH or α-Tubulin was used as the loading control (Wang et al., 2018b).

### Statistical analysis

Data were Mean ± SEM. Statistical significance (*p* < 0.05) for all other variables was determined by one-way analysis of variance followed by a Tukey’s post hoc test.

## Data Availability

All data generated or analyzed in the present study are included in this published article and the supplementary material. Requests for materials should be addressed to the corresponding author.

## Abbreviations

± dL/dt: Maximal velocity of shortening/relengthening
BECN: Beclin1
BM: Bone marrow
FFI: Fura-2 fluorescence intensity
FITC: Fluorescein isothiocyanate
KHB: Krebs-Henseleit bicarbonate
LAD: Left anterior descending coronary artery
LV: Left ventricular
LVEDD: Left ventricular end-diastolic diameter
LVESD: Left ventricular and end-systolic diameter
MI: Myocardial infarction
MSCs: Mesenchymal stem cells
mTOR: Mammalian target of rapamycin
PBS: Phosphate-buffered saline solution
PCI: Percutaneous coronary intervention
PS: Peak shortening
TEM: Transmission electron microscopy
TPS: Time-to-PS (TPS)
TR_90_: Time-to-90% relengthening
WT: Wild-type
